# In this issue: Volume 115, Issue 3, March 2024

**DOI:** 10.1111/cas.16115

**Published:** 2024-03-07

## Anti‐angiogenesis in colorectal cancer therapy

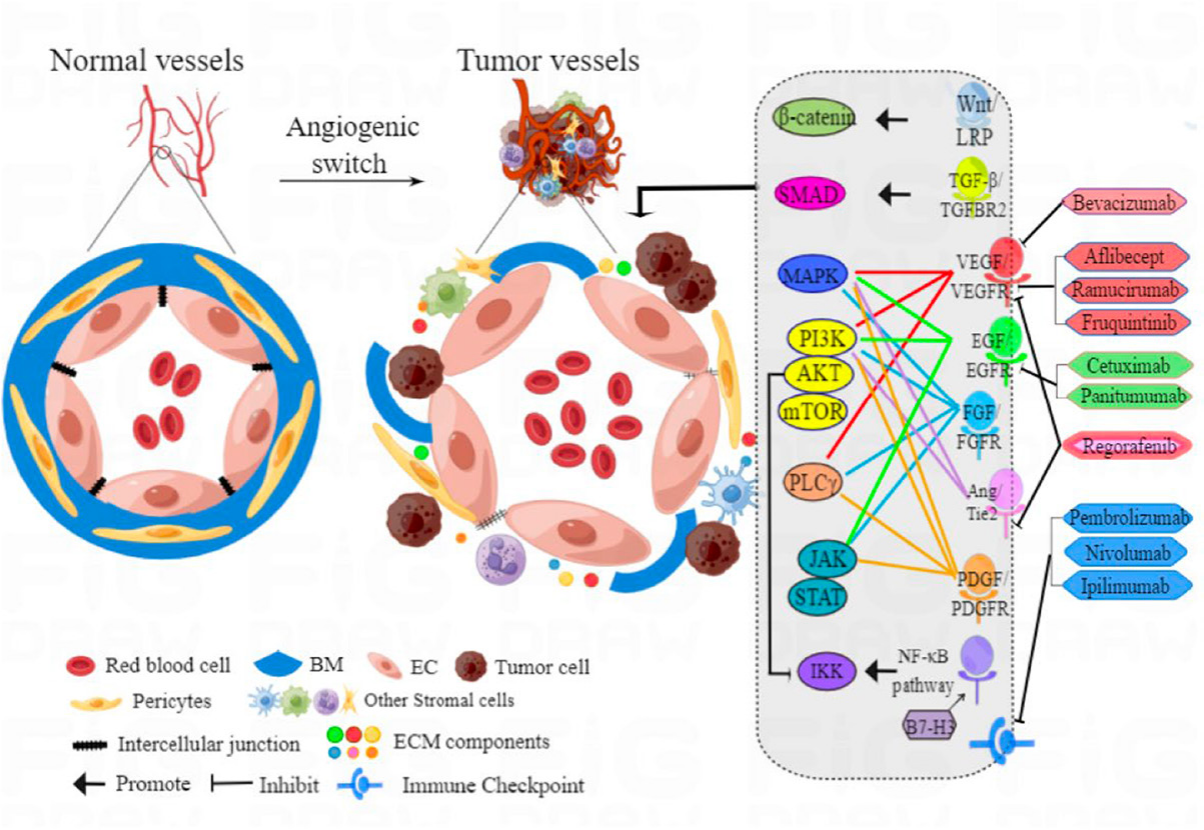

Colorectal cancer (CRC) is the third most common type of cancer worldwide, making it a significant health concern. This type of cancer generally begins with the abnormal growth of cells in the colon or rectum and may eventually transform to a malignant cancer that spreads to other organs. The key to the progression of CRC lies in a process called angiogenesis, where new blood vessels form to supply essential nutrients and oxygen to the tumor. Notably, these blood vessels have unique characteristics that differentiate them from other blood vessels. Targeting the pathways involved in angiogenesis is emerging as a popular therapy for treating CRC.

Now, scientists have reviewed the available information on anti‐angiogenesis therapy for CRC to increase awareness and facilitate further research on the subject. The review presents a detailed account of various aspects related to CRC. This includes elucidation of the role of RNA molecules that can potentially serve as diagnostic markers, as well as the influence of gut bacteria on inflammation, immunity, and response to CRC treatment.

Various treatment strategies for CRC are discussed in the review. These include surgery, chemotherapy, targeted therapy, immunotherapy, and radiotherapy. Targeted therapies based on anti‐angiogenesis that focus on specific biomolecules to hinder blood vessel formation, such as bevacizumab and aflibercept or cetuximab and panitumumab, which target different molecules involved in CRC progression. Till now, anti‐angiogenesis agents have demonstrated significant advantages in terms of their therapeutic efficacy over conventional treatments.

The authors also discuss that in response to the potential threat of resistance to certain drugs, we must explore immunotherapy, a promising method that enhances the body's immune system to fight CRC. Specifically, pembrolizumab and nivolumab are stressed as potential treatments, as they prevent the cancer cells from evading the immune system.

Despite these advancements, challenges in treatment include overcoming drug resistance and understanding the complex interactions in the tumor environment. Researchers are actively exploring ways to target tumor blood supply, enhance the body's natural defenses, and improve treatment outcomes. Addressing these challenges is crucial for more effective and personalized CRC treatments in the future.

https://onlinelibrary.wiley.com/doi/full/10.1111/cas.16063

## Circular RNA circWNK1 inhibits the progression of gastric cancer via regulating the miR‐21‐3p/SMAD7 axis

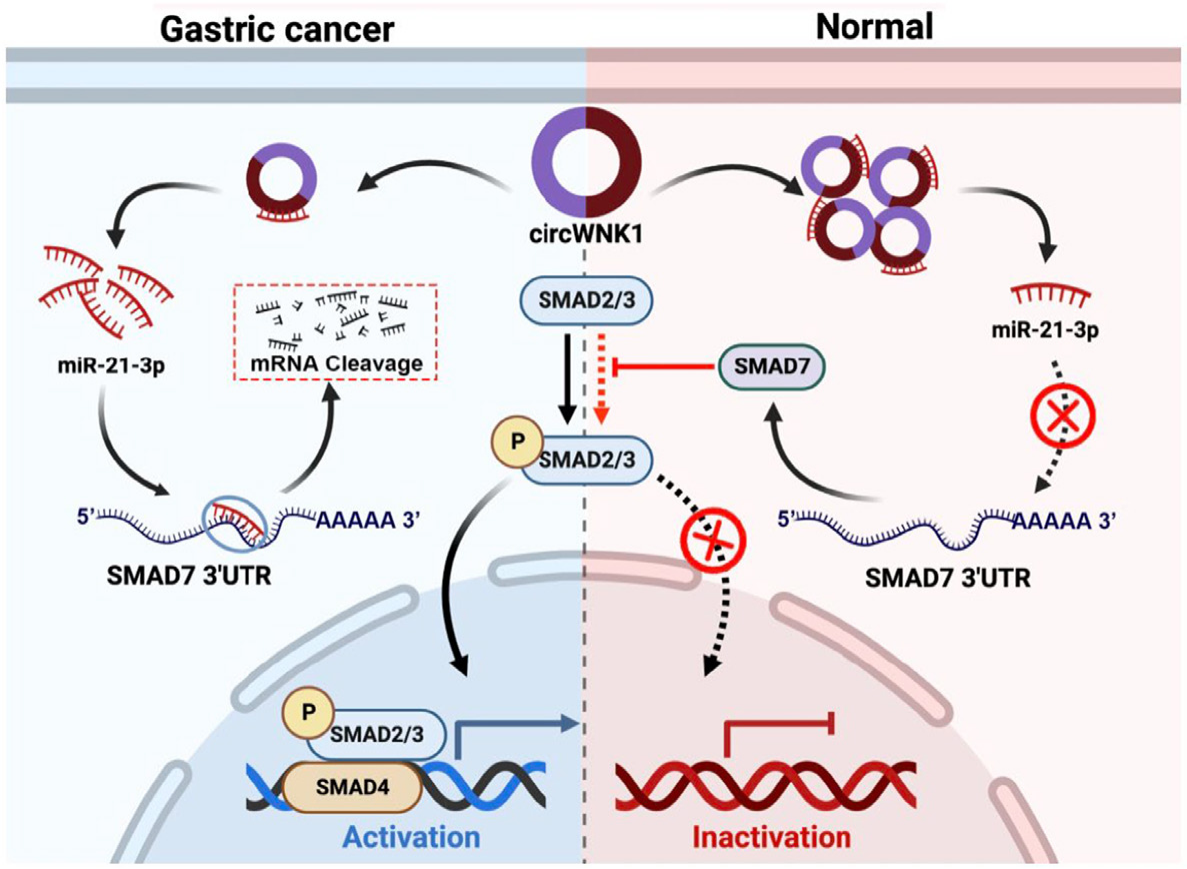

Gastric cancer (GC) is a highly aggressive form of cancer characterized by a complex mix of genetic changes and dysfunctional molecular pathways. This makes the development of effective treatments quite challenging, especially for advanced stages of the disease. Recent research has revealed the potential involvement of circular RNAs (circRNAs) in cancer development, notably in GC.

Because of their structural configuration, circRNAs are more resistant to degradation. Their stability helps them play several important roles in regulating cellular functions. However, comprehensive insights into the mechanisms through which circRNAs influence the development and progression of GC are still lacking.

Now, scientists have employed cutting‐edge molecular techniques to uncover more pieces of the puzzle underlying GC development and progression. They found that the levels of a specific circRNA called hsa_circ_0003251, also known as circWNK1, were lower in GC tissues than in normal tissues.

Upon further investigation, they found that circWNK1 acts as a guardian, inhibiting the proliferation of GC cells. It does so by limiting the ability of GC cells to move and invade surrounding tissues. In addition, the researchers revealed that circWNK1 exerts inhibitory effects on certain cell processes involved in GC progression, including migration, invasion, and epithelial–mesenchymal transition, which are associated with cancer spread.

Overall, circWNK1 appears to be a natural GC suppressor, keeping the cancer cells in check. circWNK1 could be a potential biomarker for diagnosing GC and devising targeted therapies to address tumor‐specific behaviors.

https://onlinelibrary.wiley.com/doi/10.1111/cas.16067

## Patient survey on cancer genomic medicine in Japan under the national health insurance system

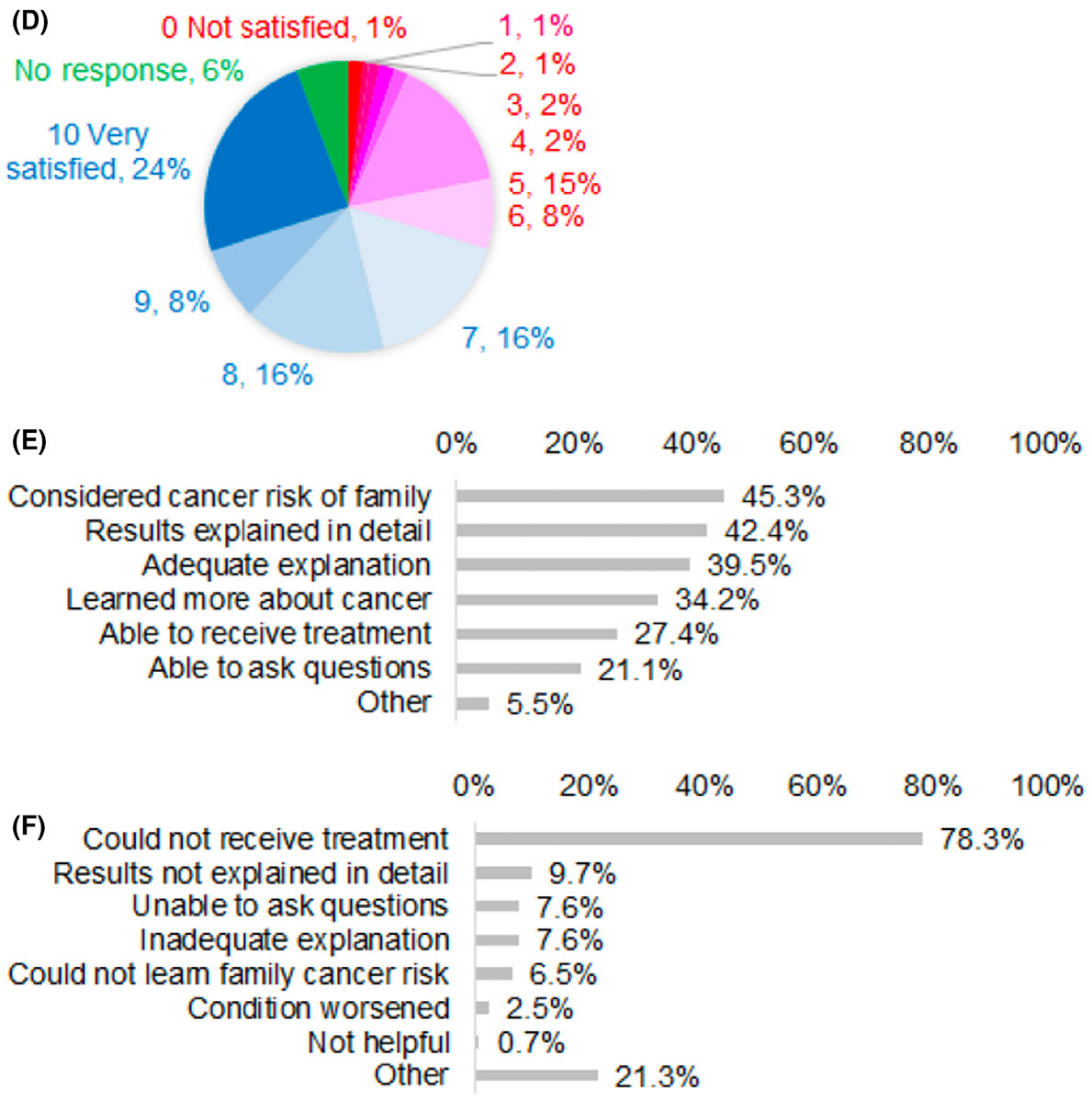

Genetics can play an important role in the development of cancer. Certain genes, called oncogenes and tumor suppressor genes work together to regulate cell division and growth. Mutations in these genes can potentially cause uncontrolled cell growth, leading to the development of cancer. Some cancers have a strong hereditary component, while others result from exposure to cancer causing substances during a person's lifetime.

Comprehensive cancer genomic profiling (CGP) is a powerful tool used to study the genetic information from cancer cells. In Japan, CGP tests are a part of the national health care system for patients completing the standard cancer treatment. CGP can help identify specific alterations in genes, detect the cancer type, and develop new specific treatment strategies. Many patients take CGP tests after standard treatment, but only a few receive new treatments. A lack of understanding about CGP tests can have a negative effect on the psychology of patients and their families. Hence, it is important to understand the effects of the CGP test process on the psychology of patients and their families.

To find out patients' contentment and issues with respect to the CGP test process, scientists conducted a survey based on a questionnaire to find out the patients' source of information on CGP tests and their level of satisfaction.

They discovered that most of the patients were introduced to CGP tests by healthcare professionals and provided informed consent to their physicians. Additionally, the most common concern of half of the participants was lack of access to new treatment after the test results. Moreover, many patients had no access to the recommended treatment due to the lack of availability of clinically approved drugs. Overall, 64.5% of patients were highly satisfied with the CGP test process.

This first‐of‐its‐kind nationwide survey highlights the fact that the CGP test process is highly satisfying for most patients. However, measures to improve the knowledge of patients and their families on CGP tests and to ensure that more patients receive matched therapy after CGP tests are needed.

https://onlinelibrary.wiley.com/doi/full/10.1111/CAS.16065

